# Pediatric Epilepsy Surgery: The Noninvasive Presurgical Evaluation

**DOI:** 10.1159/000548477

**Published:** 2025-09-13

**Authors:** Deepankar Mohanty, Michael Quach

**Affiliations:** Department of Pediatrics, Section of Neurology and Developmental Neuroscience, Texas Children’s Hospital, Baylor College of Medicine, Houston, TX, USA

**Keywords:** Drug-resistant epilepsy, Epilepsy surgery, Neuroimaging, Neurophysiology, Functional mapping

## Abstract

**Background:**

Drug-resistant epilepsy is a debilitating condition that afflicts individuals across all demographics, including children. The only recourse for many of these individuals is neurosurgery to reduce seizure burden, by either resecting or ablating the cerebral source or modulating it with a stimulator device. In either case, a thorough presurgical evaluation is required to identify brain regions of interest and construct an appropriate surgical plan. The scope of this evaluation has grown rapidly over the years as new and refined techniques have emerged. The aim of this article was to condense the most salient points regarding investigational tools used commonly in this process and provide a framework from which epilepsy management providers can tailor their own epilepsy surgery pathway.

**Summary:**

This article will discuss criteria to identify appropriate candidates for epilepsy surgery, as well as various techniques that are used to localize seizure onset, interictally active areas, dysfunctional regions, and eloquent cortex. Topics reviewed include neuroimaging (MRI, PET, SPECT), electrophysiology (EEG and MEG), and functional mapping procedures (fMRI, TMS, neuropsychologic evaluation, intracarotid amobarbital test).

**Key Messages:**

A comprehensive, multimodal presurgical evaluation including imaging, electrophysiology, and functional mapping is essential to establish the bounds of the epileptogenic zone in relation to eloquent cortex.

## Introduction

Epilepsy is one of the most prevalent neurologic diseases, afflicting individuals across all ages and demographics with recurrent, unprovoked seizures [1]. Approximately 1/150 children are diagnosed in the first 10 years of life [2], and despite pharmacologic advancements, a significant proportion continue to suffer breakthrough seizures despite adequate dosing of two or more anti-seizure medications. This cohort is defined as having medically refractory or drug-resistant epilepsy (DRE), also referred to as intractable epilepsy. Uncontrolled seizures lead to reduction in quality of life, recurrent hospital visits, and an increase in morbidity and mortality [3].

Epilepsy surgery is typically targeted at patients with DRE to disrupt the regions and/or networks in the brain that are producing seizures. This can be done either physically (with resection or tissue ablation) or electrically (via neuromodulation). In either case, proper patient selection and a comprehensive presurgical (noninvasive) evaluation are essential to produce optimal postoperative outcomes. Providers engaging in epilepsy surgery would thus benefit from familiarity with this process. This article will review patient identification for epilepsy surgery referral, currently recommended components of the presurgical noninvasive evaluation, and strategic tailoring of these components based on patient characteristics.

## Patient Identification for Surgery

Early identification of appropriate patients for entry into the epilepsy surgical pipeline is essential. Delays in surgical referral of DRE patients can lead to worsened quality of life, increased hospital and emergency room visits, and heightened risk of sudden death in epilepsy (SUDEP) [4]. Epilepsy surgery is also likely to be more cost-effective in this cohort than prolonged medical management [5]. Reasons for delay include absence of clearly defined lesions on initial magnetic resonance imaging (MRI), older age at epilepsy onset, a slow progression to clearly defined DRE, patient or family reluctance, membership in an underrepresented minority group, and difficulty in accessing epilepsy care (either due to physical distance or economic barriers) [4, 6, 7]. The impact of these factors can be significant – a Japanese study of 93 pediatric patients with focal cortical dysplasia (FCD) estimated that 46% of them experienced surgical delays exceeding 2 years [6]. Delays in care can have significant neurodevelopmental repercussions in children. The epilepsy provider should thus remain vigilant and have early discussions regarding surgery in DRE cases, particularly those involving the aforementioned risk factors.

Surgical intervention is typically recommended for patients with lesional focal-onset DRE, and postoperative outcomes have demonstrated seizure freedom in 50–60% of these patients [8, 9]. Non-lesional cases are not as easily identified for epilepsy surgery referral, but excellent surgical outcomes are still possible, as demonstrated in a 2024 study by Bustros et al. [10] where 66% of non-lesional patients achieved Engel Class I outcomes, compared to 78% of lesional patients. We recommend that non-lesional focal DRE is considered for a presurgical evaluation, particularly if an underlying etiology is not clearly apparent.

Generalized epilepsy has historically not been considered the domain of epilepsy surgery, other than for implantation of a vagus nerve stimulator (VNS). However, there are two factors that suggest intractable generalized epilepsy patients merit a closer look from a surgical standpoint. One is that large focal cortical abnormalities with onset at an early age can appear falsely generalized on scalp electroencephalography (EEG), and the EEG findings should not be considered a contraindication to imaging studies where suspicion exists, or to localized surgical intervention if a lesion is identified [11]. The second factor is that rapid developments in the field of neuromodulation have recently identified intracranial thalamic targets (mainly the centromedian nucleus) for the treatment of idiopathic generalized epilepsy, with initial trials showing >80% reduction in convulsive seizures at 1-year follow-up [12, 13]. The intracranial responsive neurostimulation device can also be used concurrently with VNS and may display additive synergy [14], so the presence of a VNS should not be a contraindication for further surgical investigation.

## Concepts in Epilepsy Localization: Zones and Networks

The primary purpose of the presurgical investigation is to formulate a hypothesis regarding the location of seizure onset. Classically, this has involved delineating the margins of several “zones” that define areas of epileptogenicity, functional deficit, and structural anomaly [15]. Each of the various components of the presurgical evaluation is particularly well suited to detect one or more of these regions (Table 1). The seizure onset zone (SOZ) is of obvious interest as the brain area where seizures are estimated to start, but long-term seizure reduction may also require elimination of part or all of the epileptogenic zone (EZ), which contains the regions of cortex that are recruited into the seizure as it propagates into its habitual pathway. Identification of these areas may be aided by locating structural lesions, the irritative zone (IZ) (where interictal discharges originate), the symptomatogenic zone (SZ) (where ictal semiology originates), and the functional deficit zone (dysfunctional cortical tissue that may be epileptogenic in nature). Additionally, any surgical planning requires demarcation of eloquent cortex to avoid postoperative functional deficits.

**Table 1.** The zones of interest in the epilepsy surgery evaluation (as defined by Rosenow et al. [15]) and investigative modalities that are particularly useful in delineating each one

Zones in epilepsy surgery	Definition	Useful modalities for localization
Seizure Onset Zone (SOZ)	The area of cortex from which seizures are being generated currently	EEG, MEG, PET, SPECT
Epileptogenic Zone (EZ)	The entire cortical area that is recruited into seizure production	EEG, MEG, PET, SPECT
Irritative Zone (IZ)	The region from which interictal epileptiform activity is generated	EEG, MEG
Symptomatogenic Zone (SZ)	The location from which clinical ictal symptoms originate	vEEG (semiology)
Functional Deficit Zone (FDZ)	The area of cortex that is functionally abnormal during the interictal period	Physical exam, neuropsychological testing, PET, fMRI
Epileptogenic lesion	A structural lesion that is contributing to epileptic seizure genesis	MRI, CT, PET
Eloquent cortex	The brain regions responsible for vital functions such as movement, language, and memory	fMRI, MEG, neuropsychological testing, TMS

EEG, electroencephalogram; MEG, magnetoencephalogram; PET, positron emission tomography scan; SPECT, single photon emission computed tomography; fMRI, functional magnetic resonance imaging; TMS, transcranial magnetic stimulation.

This classical view of epilepsy surgery has been augmented by research demonstrating the connectivity between spatially disparate groups of neurons – referred to as the “epileptogenic network” [16, 17]. For example, simultaneous EEG and functional MRI of generalized epilepsy patients have shown hemodynamic changes (blood oxygen level-dependent [BOLD] decrements) in the default mode network (primarily the frontal and parietal cortices and posterior cingulate) prior to EEG spike onset, suggesting coherence between propagating neuronal dysfunction and epileptiform activity [18]. Focal spikes have also been shown to be associated with transient hemodynamic changes in the wider ictal onset zone [19]. Two main avenues of thought have emerged from the network hypothesis. One is the view of seizure propagation as occurring along a series of networked “nodes,” where precise interruption of the pathway (either mechanically or via neuromodulation) between highly connected points may lead to improved seizure control [20]. The other is the recognition that epileptic discharges with high connectivity can have cognitive consequences [21], which could potentially be ameliorated with management of the underlying disease.

Both the zone and network models demand high precision from the surgical team to produce significant seizure reduction while limiting functional losses. In the upcoming sections, we will discuss the components of the presurgical workup and how they can contribute the requisite targeting data.

## Seizure Semiology

The first step in conceptualizing a localization model is a detailed history focusing on semiology of habitual seizures, including video evidence from the patient and/or family if available. If video evidence is not readily available, it can be acquired during a subsequent video-EEG (vEEG) study. Analysis of semiologic features not only is essential in early differentiation of epileptic from nonepileptic events but also aids in establishing an early diagnosis of focal or generalized epilepsy. Semiology is also uniquely qualified to delineate the patient’s SZ [22]. Certain well-studied clinical patterns can be highly localizing in this regard. Examples include the ictal pout (“Chapeau de Gendarme”) which implicates the anterior prefrontal region, the “fencing posture” implicating the supplementary motor area, and ictal hypersalivation which is typically indicative of insulo-opercular involvement [23]. An excellent primer on the lateralizing and localizing value of specific semiologic features was recently published by the International League Against Epilepsy (ILAE) and serves as recommended reading for epileptologists and neurosurgeons [23].

## Neuroimaging

### Magnetic Resonance Imaging

Imaging of the brain parenchyma is typically one of the first steps in the epilepsy surgery pathway, and an MRI is recommended for all potential candidates [24]. MRI visualization of the epileptogenic lesion has prognostic implications – children with complete resection of MRI-defined lesions have significantly higher odds of seizure freedom than those without [25, 26]. A magnetic field strength of 3 Tesla (3T) is standard for epilepsy imaging as it provides superior image resolution over 1.5T and can lead to identification of additional lesions [27]. 7T MRI is an emerging technology that is not yet widely available clinically, but has demonstrated additional utility over 3T. For example, Bubrick et al. [28] detected new lesions in over one-third of epilepsy patients that were deemed non-lesional after 3T MRI scans, and better characterized the margins of a known 3T lesion in another one-third. Wang et al. [29] also noted additional lesions using 7T in 19% of patients with known multifocal abnormalities such as polymicrogyria and periventricular nodular heterotopia. If technological improvements result in increased clinical accessibility in the future, 7T MRI may well become an integral part of the epilepsy evaluation.

Epileptogenic lesions such as FCDs can be quite subtle on imaging, so high-resolution MRI “epilepsy protocols” are required for a thorough evaluation. The ILAE Neuroimaging Task Force recommends standard inclusion of the following sequences: a millimetric resolution (1 × 1 × 1 mm^3^ voxel size) 3D T1-weighted sequence such as magnetization-prepared rapid gradient echo, a millimetric resolution 3D fluid-attenuated inversion recovery sequence, and a 2D coronal T2-weighted sequence (such as turbo spin echo) with sub-millimetric voxel resolution (0.4 × 0.4 × 2 mm^3^) to assess hippocampal anatomy [24]. Gadolinium contrast should be considered if tumors, vascular malformations, or infections are suspected. Contrast can also assist in mapping out vascular pathways for surgical planning.

The patient’s age and associated brain development should be kept in mind when reviewing brain MRIs for identifiable lesions. Brain maturation and the associated myelination of white matter lead to increased gray-white differentiation on both T1 and T2 sequences [30], which is particularly relevant in the identification of subtle cortical dysplasia and nodular heterotopia. Non-lesional MRIs performed in infants with suspected focal epilepsy should be repeated after 24 months of age (when myelination has sufficiently progressed) to ensure that no such anatomic malformations were missed on initial imaging.

Visual analysis of the MRI should be performed by trained neuroradiologists, neurosurgeons, and epileptologists, but in some cases (over 30% according to some studies [24]) this is insufficient to establish a diagnosis. Computer-aided MRI postprocessing and quantitative analysis have shown promise in increasing the yield of the epilepsy workup; Table 2 briefly discusses a few examples of recommended methods [24, 31–33]. Fully automated neural networks specializing in detection of MRI lesions are another recent development in computational neuroimaging. One such network trained to detect FCDs – the Multi-center Epilepsy Lesion Detection Graph – displayed a 64% sensitivity for dysplasias in MRI-negative patients and significantly increased positive predictive value of MRI findings [34]. Although these networks have not come into widespread clinical use so far, they show promise as a potential future tool in the management of MRI-negative epilepsy.

**Table 2.** Summary of some commonly used quantitative analytical methods in MRI for epilepsy surgery

Analysis method	Purpose	Notes on utility
Hippocampal volumetry	A systematic manual or automated assessment of the relative sizes of bilateral mesial temporal lobe structures	Increases sensitivity for detection of hippocampal atrophy [24] and predicts degree of cell loss on histopathology [31]
T2 relaxometry	T2 relaxation – the progressive decay of magnetization as spinning dipoles fall out of phase in spin echo sequences – takes longer to occur in gliotic tissue. T2 relaxometry measures regional variance in T2 relaxation time	Increased T2 relaxation times correlate with higher degree of mesial temporal gliosis in TLE and can aid in identification of the EZ [32]
VBM	Correlates anatomic models with measurements of gray and white matter intensity on a voxel basis	Aids in identifying regions where transition from gray to white matter is slow, suggesting blurring of the gray-white margin. Can increase sensitivity for FCD by up to 40% [33]

FCD, focal cortical dysplasia; VBM, voxel-based modeling.

### Positron Emission Tomography

**Fig. 1.**
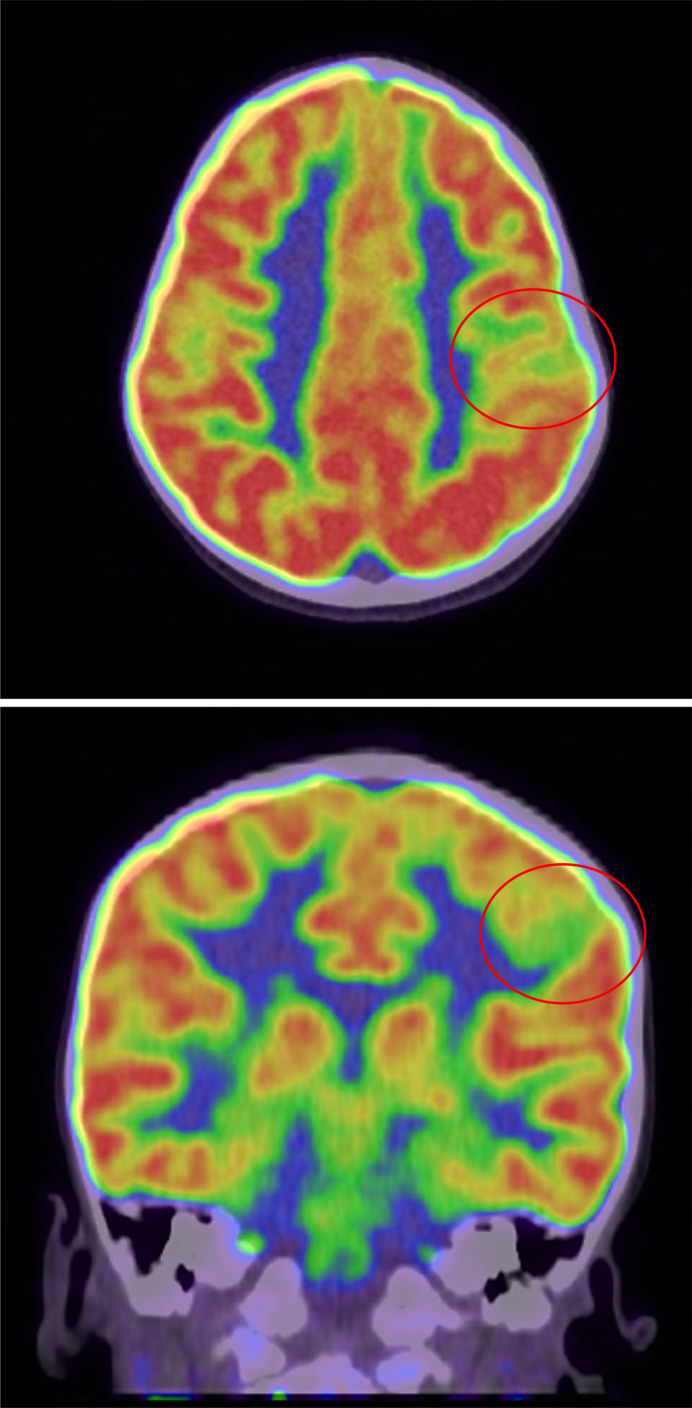
Axial and coronal views of an area of interictal PET hypometabolism in the left central sulcus and precentral gyrus, corresponding to an epileptogenic focus.

Positron emission tomography (PET) measures cerebral levels of a previously injected radio-ligand as an analog for local metabolic rate. Typically, the ligand used is ^18^F-fluorodeoxyglucose ([^18^F]-FDG, henceforth referred to as FDG), which binds to glucose in the bloodstream and can cross the blood-brain barrier. Epileptogenic regions tend to be dysfunctional and hypometabolic interictally (Fig. 1) and hypermetabolic ictally. Continuous EEG monitoring is strongly recommended just prior to and after the PET scan to establish the brain state during acquisition as ictal, interictal, or postictal [35]. Additionally, as FDG-PET relies on blood glucose transport, pre-scan hyperglycemia inconsistent with patient baseline is a contraindication [35].

PET co-registration with CT (PET-CT) or MRI (PET-MRI) is recommended for ease of visualization and improved correlation of hypometabolic areas with neuroanatomy. Use of PET-MRI in the epilepsy surgery evaluation reduces the need for invasive monitoring and improves likelihood of long-term postoperative seizure freedom [36]. Automated techniques such as voxel-wise statistical parametric mapping can be used to augment visual analysis in pediatrics as long as age-adjusted norms are used for control datasets [35].

Focal FDG-PET hypometabolism localization has been associated with favorable seizure reduction outcome in epilepsy surgery patients both with and without MRI-defined lesions. The odds ratio of favorable outcomes is heightened even further when FDG-PET localization is concordant with EEG and/or MRI findings [37]. FDG-PET should also be strongly considered as an adjunctive test in MRI-negative focal epilepsy. It can reveal previously unseen lesions or delineate the EZ via hypometabolism in the absence of lesional margins, thus altering the surgical plan in nearly 1/3 of MRI-negative or discordant cases [38].

### Single Photon Emission Computed Tomography

In epilepsy, single photon emission computed tomography (SPECT) is used to assess tissue levels of a bloodstream-injected, technetium-based radiotracer bound to a biologically active ligand – typically hexamethyl-propylene-amine-oxime or ethylene-cysteine-diethylester. The ligand allows the tracer to access past the blood-brain barrier, and accumulated levels of tracer in various brain areas can be used as an analog for regional cerebral blood flow (rCBF) and local metabolic rate. Interictally, the SOZ and EZ are likely to be hypometabolic with lower rCBF, similarly to PET findings. At the onset of a seizure, metabolic demand and rCBF are theoretically highest in the SOZ and regions of immediate propagation, allowing SPECT to identify those regions if the tracer is injected promptly [39]. Over time, seizures tend to propagate further from the SOZ and thus radiotracer injection greater than 45 s after ictal onset is more likely to lead to false or non-localizing findings [40]. Some latency between ictal onset and injection is unavoidable however, so SPECT is unlikely to be useful in patients with brief seizures lasting less than 15–20 s.

Visual analysis of SPECT can be difficult, as many areas of hypo/hyper-perfusion may appear due to physiologic variance in cerebral blood flow over time. Subtraction ictal-interictal SPECT co-registered to MRI (SISCOM) measures the relative change in rCBF and local metabolic rates between the ictal and interictal scans. As this relative change is likely to be higher in epileptogenic areas given their baseline hypometabolism and ictal hypermetabolism, localization yield from SPECT can be significantly enhanced by SISCOM [40]. This has prognostic consequences as well – Jalota et al. [41] found that resection (either complete or partial) of the region identified by SISCOM was associated with seizure-free outcome in over 50% of patients, including both temporal and extra-temporal epilepsy – making SISCOM an invaluable part of the epilepsy evaluation. Ictal-interictal SPECT analyzed by statistical parametric mapping (ISAS) is an additional technique that uses SPECTs from a control database of healthy individuals to account for physiologic variance in cerebral blood flow. This can reduce false negative or discordant data produced by SISCOM and further improve localization results [42].

## Neurologic Electrophysiology

### EEG and Electrical Source Imaging

The cerebral cortex displays laminar organization – layers of neurons are arranged in columnar fashion with parallel orientation of cell bodies and axons. Due to their parallel alignment, neighboring neurons firing synchronously from dendrite to axon create summated regional electrical activity flowing in one direction, referred to as an electrical dipole [43]. The practice of EEG relies on detecting the voltage field changes induced by these dipoles, whether locally (via intracranial electrocorticography) or more distantly on the scalp (surface EEG). Radial dipoles, where the flow of current is perpendicular to the scalp surface (such as on superficial locations on the cortical convexity) and oriented toward the overlying electrode, are maximally detected on surface EEG. For the same reason, tangential dipoles (where current flow is parallel to the scalp surface, such as within sulcal walls) are poorly detected.

Surface EEG electrodes are deployed around the scalp with anatomical measurements via the “10–20” system, and the standard setup consists of about 25 electrodes. High-density EEG (HD-EEG) arrays also exist with closer electrode spacing, allowing for anywhere from 64 to 256 electrodes. Outpatient vEEG monitoring is typically one of the first tests performed after an individual has unprovoked seizures. These short studies often capture interictal epileptiform discharges (IEDs) arising from the IZ but typically do not record seizures unless ictal burden is very high. IEDs are generally negative in polarity and are classified into either spikes (waveforms with a duration under 70 milliseconds) or sharp waves (duration 70–200 milliseconds). They are caused by rapid depolarization shifts in groups of cortical neurons, and after-going slow waves (when noted) represent the local hyperpolarization in response. Focal IEDs are a strong indicator of underlying focal epilepsy and can be suggestive of the SOZ, particularly if the brain region responsible for the clinical semiology of the seizure (the SZ) is concordant with the IZ. Specific types of IEDs are particularly relevant in certain types of epilepsy [44] and will be discussed briefly here:

1. Anterior temporal spikes are seen in 90% of patients with mesial temporal lobe epilepsy (TLE) and are highly evocative of that condition, particularly in combination with concordant temporal intermittent rhythmic delta activity.
2. At least one-third of patients with mesial TLE have independent bitemporal IEDs, reflecting either rapid propagation from a unilateral source or bilaterally independent spike generators. Many patients can still benefit from unilateral resection targeting the more interictally active temporal lobe, so the proportion of right- versus left-sided discharges should be determined and bitemporal IEDs should not be considered a contraindication to surgery.
3. In focal occipital epilepsy, lateral spike sources are typically detected in occipital or posterior temporal electrodes, but in mesial cases can be seen in bilateral or even contralateral occipital contacts due to rapid propagation.
4. Caution should be exercised in dismissing a focal hypothesis with generalized-appearing IEDs if the described ictal semiology seems frontal lobar in origin (such as asymmetric tonic posturing or distal stereotypies) [45]. Focal discharges arising from mesial or basal frontal cortices may appear falsely diffuse on surface EEG due to rapid bi-hemispheric propagation and the significant distance between source and detector, which must be traversed via volume conduction.
5. Focal discharges from spatially limited sources distant to scalp (such as insula, basal temporal region, or operculum) may not be visible on surface EEG at all or present only as projected slow waves.

Although significant information regarding the IZ can be gathered from IEDs, the IZ and SOZ can sometimes be discordant [46]. Thus, vEEG recording of ictal onset is essential for the epilepsy surgery evaluation to confirm localization of the SOZ. Prolonged vEEG monitoring in the epilepsy monitoring unit is usually required; at least 3 days of continuous EEG is recommended, and 4–6 days is optimal in most cases [47]. Shorter duration may suffice for particularly refractory patients with daily seizures, but if a patient has multiple seizure types, they should all be characterized prior to surgical planning as they could arise from independent SOZs. Ictal EEG analysis should be focused on the immediate peri-onset period prior to seizure propagation. Ictal onset patterns can be variable [44]; some common examples are discussed in Table 3. Higher frequency patterns are generally lower voltage and propagate less distance than low-frequency waveforms; their presence on an ictal scalp EEG is often indicative of superficial neocortical onset [44]. Video review of seizures is also vital as clinical ictal semiology can reveal the SZ, and the onset of clinical signs prior to any change in EEG signature would suggest a distant mesial, basal, or insular source not well detected on scalp.

**Table 3.** Common EEG patterns seen at ictal onset in both surface EEG and intracranial EEG data, along with descriptions and noted associations

EEG pattern	Description
LVFA	Fast activity ranging from 15 to 30 Hz in mesial temporal seizures and 30–100 Hz in neocortical seizures
	LVFA can be seen on surface EEG but is more commonly recorded on intracranial EEG
Pre-ictal spiking with low-frequency rhythmic spikes followed by LVFA	Rhythmic spikes or spike waves in the delta frequency range that transition to LVFA, often accompanied by electro-decrement
Polyspike burst followed by LVFA	High-amplitude polyspikes in a high-frequency (beta range typically) burst that transition to LVFA
Low-frequency rhythmic spikes or spike-wave complexes	Rhythmic spikes or spike-wave complexes in the delta range (1–4 Hz)
	Slower rhythmic patterns tend to be seen more frequently in epilepsy associated with neurodevelopmental tumors
Rhythmic theta/alpha sharply contoured activity	Rhythmic spikes or sharp waves typically occurring at a theta or alpha (5–12 Hz) frequency
	A notable example is the 5–9 Hz rhythmic spikes in temporal lobe characteristic of seizure onset in mesial temporal epilepsy
Slow wave or DC shift followed by LVFA	High-amplitude slow wave in low delta range (0.5–2 Hz) or DC shift characterized by frequency below 0.5 Hz (also known as infraslow activity) that evolves to LVFA

LVFA, low-voltage fast activity; DC, direct current; Hz, hertz.

**Fig. 2.**
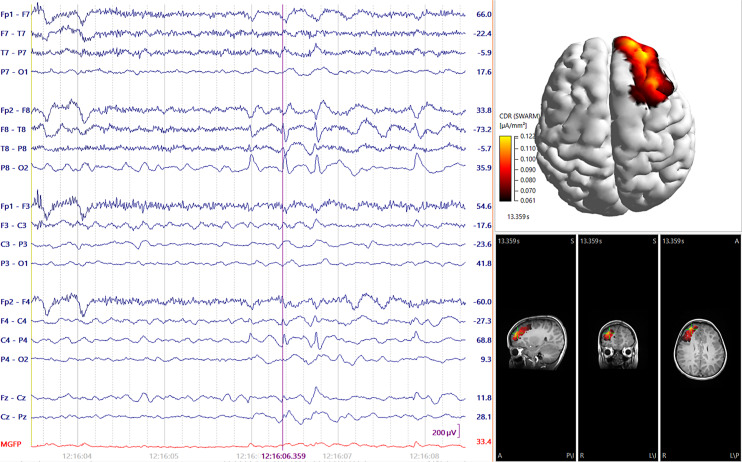
SWARM analysis of a right fronto-temporal spike on EEG (left) localized to the right anterior frontal lobe (right), as indicated by modeled areas of highest current density (colored bright orange and yellow). sLORETA, standardized low-resolution brain electromagnetic tomography; SWARM, sLORETA-weighted accurate minimum norm.

Electrical source imaging (ESI) offers a quantitative method to augment visual EEG analysis. Intracranial dipole sources generate shifts in the electric field at the scalp surface which are detected on EEG. ESI uses these surface EEG signals to reverse engineer a plausible cerebral source which could have generated them, referred to as the “inverse solution” (in contrast to the “forward solution” which determines the scalp correlate of a known intracranial source). This analysis can be performed by various methods. One is equivalent current dipole (ECD) modeling, which computes a single focal dipole source that could have generated the detected surface signal. Multiple intracranial dipole locations can lead to identical surface EEG correlates however, so the results must be interpreted carefully within context of the remainder of the clinical data. Another form of analysis relies on distributed source modeling algorithms such as standardized low-resolution brain electromagnetic tomography or standardized low-resolution brain electromagnetic tomography-weighted accurate minimum norm, which can generate a current density map of the cortical surface at a moment of time based on available EEG data (Fig. 2). A retrospective study by Russo et al. [48] has shown that standard surface EEG ESI accurately predicted the surgical resection margin in the majority of cases. Wanders et al. [49] also demonstrated that ESI using HD-EEG data is capable of even higher localization accuracy and predicted seizure freedom in the majority of pediatric FCD patients when the ESI-detected location was resected.

### Magnetoencephalography and Magnetic Source Imaging

Any electrical source generates both an electrical field and a perpendicularly oriented magnetic field. Magnetoencephalography (MEG) uses superconducting quantum interference devices to detect magnetic field changes on the scalp induced by intracranial electrical dipoles. This technique has an advantage over surface EEG – magnetic waves are not as significantly attenuated by passing through human tissue as electrical signals. MEG can thus detect focal spikes with low surface area which activate as little as 3–6 cm^2^ of neocortex, whereas EEG can require 10 cm^2^ of activation [50]. Source localization is also aided by a higher temporal resolution (less than 1 millisecond) and larger volume of sensors (typically over 300) than standard EEG, though HD-EEG can close some of the gap in that regard. Due to the perpendicular orientation of electrical and magnetic fields, MEG is particularly well suited to detect tangential dipoles such as those arising from sulcal walls or regions of abnormal anatomy. EEG remains superior at detecting purely radial dipoles, which makes the two techniques highly complementary to each other in source localization. Disadvantages of MEG include equipment expenses and maintenance – the superconducting quantum interference devices are only operable within a few degrees of absolute zero and need to remain cooled by liquid helium. Cerebral magnetic fields are also several orders of magnitude weaker than those generated by the Earth and external electrical equipment, so a triple-layer magnetically shielded room is necessary to eliminate signal artifacts. MEG sensors are usually placed in a helmet rather than adhered to the scalp like EEG, so patient motion can cause significant degradation of the signal. MEG studies are thus limited in duration (less than 2 h in most cases), and the likelihood of capturing seizures is low. In rare cases when this is achieved, the ictal data can be quite helpful – resection of ictal MEG-localized SOZ is associated with increased likelihood of postoperative seizure freedom [51].

**Fig. 3.**
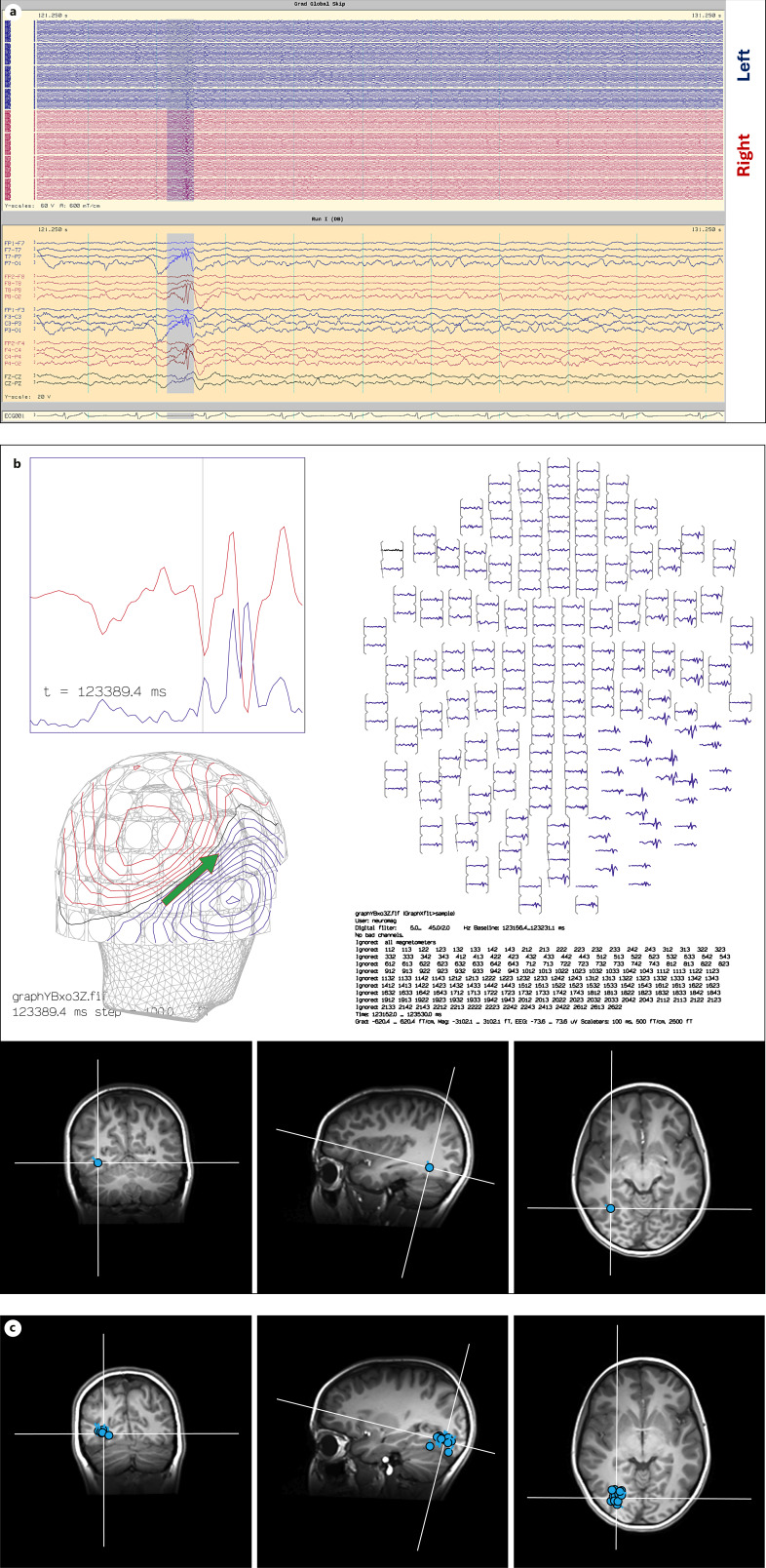
Localization of interictal spikes on MEG using the equivalent current dipole (ECD) method. **a** An interictal polyspike is detected with highest amplitude on the right EEG sensors (O2 > O1) below, and in the right occipital MEG sensors above. **b** Source analysis of the initial waveform of the discharge localizes to an ECD in the basal temporal region. **c** Subsequent analysis of multiple interictal spikes within the recording demonstrates consistent localization to the basal temporo-occipital area, forming a dipole cluster.

EEG reading is typically dependent on visual analysis of waveforms, but MEG reading is not, due to high channel density and the need to mathematically reconstruct an electrical source from a magnetic signal. Magnetic source imaging (MSI) is used to calculate ECDs in most cases and is analogous to ESI as previously discussed. MEG/MSI is highly recommended as a supplementary test in the epilepsy surgery evaluation as it frequently provides nonredundant information and can completely alter the surgical plan in some cases [52]. Identification of dipole clusters – restricted regions of cortex with a high density of localized ECDs – is particularly important (Fig. 3). Intracranial EEG sampling and resection/ablation of MEG dipole clusters (“cluster-ectomy”) have been associated with a significant increase in likelihood of postoperative seizure freedom [53, 54]. The ten most common evidence-based indications where MEG findings can be pivotal in the epilepsy surgery evaluation were identified by Bagic et al. [55] as follows:

1. Imprecise hypothesis regarding seizure onset.
2. Non-lesional MRI with suspected mesial temporal onset.
3. Multiple MRI-identified brain lesions.
4. Large brain lesions with epileptogenic subregions.
5. Patients with preexisting skull defects.
6. EEG findings that appear bilateral or generalized in patients with other indicators of focal-onset epilepsy.
7. Suspected seizure onset within the sylvian fissure.
8. Suspected interhemispheric seizure onset.
9. Suspected insular seizure onset.
10. EEG without detected epileptiform activity.

MEG also has utility as a tool for functional mapping, which will be discussed in the upcoming section.

## Functional Mapping

### Functional MRI

Functional mapping of eloquent cortex is essential for surgical planning. fMRI is one of the most used tools for this purpose in pediatrics. Regional cerebral activation during a functional task leads to an increase in local blood flow and oxygen concentration. fMRI tracks the magnetic marker of oxygenated hemoglobin to generate a BOLD signature map of the brain. Functional mapping is performed by asking the subject to perform a language, motor, or visual task and measuring BOLD activity in response. The maximal region of BOLD activation during the task would thus demarcate the primary motor cortex, dominant language hemisphere, or visual cortex. Item encoding and retrieval tasks have also been used for memory lateralization prior to surgery in TLE; this achieves comparable results with the Wada-Milner test when a varied set of items is used [56].

The temporal resolution of fMRI is on the order of seconds as it is dependent on blood flow, and BOLD changes can be localized within 2–3 mm of the site of neuronal activity [57]. fMRI is not ideal for regions with poor vascular supply, such as previously infarcted tissue. fMRI also requires the patient to remain motionless during acquisition, which often necessitates sedation in young children. This can make it difficult to test functions that depend on patient interaction, such as language or vision. Comprehensive language testing also requires task protocols in various common regional languages for multilingual patients.

### Magnetoencephalography

In addition to epileptiform source localization, MEG can also be used as a tool for functional mapping. This involves localization of expected evoked potentials associated with a functional task. Examples include somatosensory-evoked potentials in the postcentral gyrus after median nerve stimulation, motor-evoked potentials in the precentral gyrus prior to volitional finger movement, and auditory-evoked potentials in the superior temporal gyrus after an auditory tone. Language lateralization can be performed by measuring the number of valid peri-sylvian sources in each hemisphere during an expressive and/or receptive language task and subsequently calculating a “lateralization index” (LI). The LI is calculated by dividing the difference between the number of valid language sources in the right hemisphere (LR) and the left hemisphere (LL) by their sum:

$$LI=\left(LR-LL\right)/\left(LR+LL\right)$$

An LI value less than −0.1 indicates left hemispheric language dominance, LI greater than +0.1 indicates right hemispheric language dominance, and a value between −0.1 and +0.1 indicates bilateral activation without a clear dominant hemisphere. MEG language lateralization has displayed an 80–95% correlation with both the Wada test and mapping via direct cortical stimulation and has the advantage of being a fully noninvasive procedure [58]. Like fMRI, MEG benefits from patients remaining relatively still during the procedure, which often entails sedation in young children and can make interactive functional tasks challenging.

### Neuropsychological Evaluation

Comprehensive neuropsychological evaluation serves multiple essential functions prior to epilepsy surgery [59]:

1. It establishes a functional baseline in various domains of cognition and behavior against which postsurgical outcomes can be compared.
2. The evaluation helps determine domains of weakness presurgically that may not suffer significant added deficit even if eloquent cortex is resected, as well as domains of strength that should be avoided if possible.
3. Lateralized cognitive deficits observed during testing can help identify the functional deficit zone.
4. Post-evaluation counseling with the neuropsychologist assists patients and families with understanding operative risks to functional domains which can alter their decision-making.

For all these reasons, the ILAE Neuropsychology Task Force strongly recommends comprehensive pre- and postsurgical neuropsychology evaluations for all patients in the epilepsy surgery pathway [59]. Of note, care should be taken to perform presurgical evaluation at a time when patient is at baseline and not ill, postictal, or under the influence of additional temporary sedative medications beyond their maintenance regimen. If a high risk of postoperative cognitive decline is expected but cannot be avoided, cognitive rehabilitation prior to surgery can assist the patient in developing coping strategies to handle predicted deficits [60].

### Transcranial Magnetic Stimulation

Transcranial magnetic stimulation (TMS) uses extracranial magnetic fields (which can pass through bone and tissue without significant attenuation) to generate perpendicularly oriented intracranial electric fields and subsequent cortical neuron activation [61]. This has various applications within neurophysiology, but the main relevant uses for TMS within the epilepsy surgery evaluation are functional mapping of motor cortex and language regions. Motor mapping is performed via point stimulation of the precentral gyrus, central sulcus, and paracentral lobule with simultaneous EMG monitoring of contralateral upper and lower extremities. Local activation leading to reproducible EMG response validates that the target is involved in motor function. Language testing entails stimulation of postulated language areas, while the patient performs an interactive language task, such as object naming. Failure of the task entirely or increase in response latency compared to baseline would both constitute confirmatory results. Disadvantages of TMS include an inability to access mesial, basal, or sulcal regions of cortex due to limited range of the induced electric field, as well as a potential risk (albeit very low) of stimulation-induced seizure [62].

### Intracarotid Sodium Amobarbital Procedure (Wada-Milner Test)

The Wada-Milner test involves intravascular injection of sodium amobarbital to induce, in effect, hemispheric anesthesia, at which point functional testing can be performed to lateralize language and memory. If either function is dependent on the deactivated hemisphere, then associated testing results will suffer significantly compared to baseline. However, the test is rarely used in children due to difficulty in securing patient cooperation during an awake procedure with uncomfortable side effects. Functional language lateralization via fMRI has gradually proven to be highly concordant with the Wada-Milner test [63] and is the de facto modality of choice in pediatrics at this point.

## Evaluation Strategies

So far, we have discussed the more commonly used techniques to map the EZ and eloquent cortex for epilepsy surgery planning. However, the entire battery of presurgical testing described above does not necessarily need to be applied to every candidate [64]. A well-defined epileptogenic lesion on MRI located outside of eloquent cortex with concordant EEG ictal data may not need any further testing prior to surgery. However, we recommend that patients with MRI-negative focal epilepsy, discordant findings during the epilepsy evaluation, or multifocal epilepsy syndromes such as tuberous sclerosis should undergo at least epilepsy monitoring unit EEG, 3T MRI, fMRI, PET, MEG, and neuropsychological evaluation. Ictal-interictal SPECT should be considered when PET is insufficient for localization as long as seizures are reasonably frequent, reliably detectable at onset (clinically or on EEG), and have sufficient duration for injection (at least 25–30 s).

7T MRI (if available) and genetic testing should also be considered in MRI-negative patients. Individuals with generalized epilepsy undergoing consideration for a VNS or thalamic neuromodulatory device likely only need an EEG to verify the diagnosis and an MRI to rule out any cerebral lesions that could generate falsely diffuse EEG findings and guide thalamic placement if indicated. Source localization analysis (ESI or MSI) of ictal data should be strongly considered in cases where the visual EEG/MEG signature of seizure onset is poorly localizing. Deep brain lesions such as hypothalamic hamartoma do not necessarily require neurophysiology data as their seizures often do not have surface EEG/MEG correlate at onset. In terms of adjunctive functional testing, the Wada-Milner test can be valuable in carefully selected cases of developmentally mature adolescents with temporal lobe pathology and concerns for postoperative memory deficits.

After the evaluation has been completed, discussion of the case at a multidisciplinary patient management conference involving epilepsy, neurosurgery, neuroradiology, nuclear medicine, neuropsychology, administrative staff, and case management is considered standard practice at many epilepsy centers. The patient management conference improves inter-rater reliability on surgical recommendations between institutions and is highly recommended in order to adequately weigh all available data prior to surgical decision-making [65].

## Conclusion

Epilepsy surgery comes at the end of a long road for patients suffering with DRE. Care should be taken that the evaluation along the way is appropriate and accurate, to best mitigate the risk for poor seizure outcomes, functional deficits, or repeated surgery. The techniques discussed in this article form the basis from which a comprehensive evaluation can be tailored on an institutional and patient basis that will ideally lead to surgical success.

## Conflict of Interest Statement

The authors have no conflicts of interest to declare.

## Funding Sources

This article was not supported by any sponsor or funder.

## Author Contributions

D.M. gathered literature sources for review, drafted the initial text of the manuscript, amended the text after review, and approved the final version for publication. M.Q. assisted in gathering literature sources, provided critical review, and edited the body of the text for intellectual content.
